# Association Study Between Polymorphic Loci in Cholesterol Metabolism Pathway and Gallstone in the Tibetan Population

**DOI:** 10.3389/fgene.2022.902553

**Published:** 2022-05-16

**Authors:** Lifeng Ma, Hui Chen, Zhiying Zhang, Lijun Liu, Yiduo Zhao, Yansong Li, Zhipeng Zhao, Haitao Chen, Longli Kang

**Affiliations:** ^1^ Key Laboratory for Molecular Genetic Mechanisms and Intervention Research on High Altitude Disease of Tibet Autonomous Region, Xianyang, China; ^2^ Key Laboratory of High Altitude Environment and Genes Related to Diseases of Tibet Autonomous Region, School of Medicine, Xizang Minzu University, Xianyang, China; ^3^ School of Public Health, Sun Yat-sen University, Shenzhen, China

**Keywords:** cholesterol, single nucleotide polymorphism, FXYD2, Tibetans, gallstones

## Abstract

**Background:** The incidence of gallstones in the Tibetan population is increasing rapidly. Previous studies indicated that genetic variation located in the cholesterol metabolism pathway may be associated with the incidence of gallstones.

**Methods:** By recruiting 132 Tibetan gallstone patients and 52 normal Tibetan controls, we performed next-generation sequencing for 508 genes in the cholesterol metabolism pathway. Additionally, by integrating the sequence data of 41 normal Tibetan subjects in the public database, we finally obtained 93 normal Tibetan controls. Single nucleotide polymorphisms (SNPs) calling were performed by using the GATK pipeline. The quality control criteria for SNPs were: missing rate <0.05; minor allele frequency (MAF) > 0.01; and *p* value >0.001 in the Hardy-Weinberg Equilibrium (HWE) test. To eliminate the influence of population heterogeneity, Principal Component Analysis (PCA) was carried out by using the smartpca software. Association analyses were performed by Plink software. Multiple tests were adjusted by the false discovery rate (FDR) method.

**Results:** A total of 2,401 SNPs were obtained by analyzing 508 genes, and 2,011 SNPs left after quality control. After adjusting the eigen vectors, we found that 10 SNPs (SNV05997, rs80145081, rs80005560, rs79074685, rs748546375, rs201880593, rs142559357, rs750769471, rs869789 and rs4072341) were significantly associated with gallstone. Subsequently, by comparing the case group with our control group and the public database control group separately, we further found that the SNP rs869789 was consistently significantly associated with gallstone (*p* = 9.04 × 10^–3^ in cases vs. our controls and 5.73 × 10^–3^ in cases vs. public controls, respectively).

**Conclusion:** By systematically analyzed SNPs in the cholesterol metabolism pathway, we identified one polymorphic locus rs869789 significantly associated with the pathogenesis of gallstone in the Tibetan population. This study will provide clue for further mechanism study of gallstone in the Tibetan population.

## Introduction

Gallstone disease (GD) is the most common gallbladder disease (Pang et al., 2020), affecting 10–20% adult worldwide. The incidence of gallstone increased sharply during the past decades. Complications of GD include cholecystitis, pancreatitis, and so on. And, it can also significantly increase the risk of cardiovascular disease, finally leading to serious public health problems (Hu F. et al., 2021). In addition to bile acids, other components of bile including cholesterol, fatty acids, phospholipids and bilirubin (Grigor’eva and Romanova, 2020). Cholesterol stones account for more than 80% of gallstones (Hu F. et al., 2021). Most gallstones are caused by the excessive and rapid mobilization of cholesterol into the bile through the liver. The high secretion of free cholesterol in the bile leads to the oversaturation of cholesterol, resulting in the accelerated crystallization of cholesterol in the gallbladder bile, the excessive secretion of bile mucin, and the weakening of gallbladder power, and finally the formation of stone core (Di Ciaula et al., 2019; Grigor’eva and Romanova, 2020; Sun et al., 2021). In general, the formation of GD is due to the imbalance of cholesterol, bile acid, lecithin, and other components in the bile.

GD is a complex disease, and its main pathogenic factors include living environment, eating habits and genetic background (Di Ciaula et al., 2018). Studies have shown that the risk of gallstones in East Asian countries including China is lower than other areas (Lammert et al., 2016), however, the incidence rate of gallstones in Qinghai-Tibet Plateau is high. Tibet is located in the southwest frontier of China, with an average altitude of more than 4,000 m and a thin oxygen content. Hypoxia inducible factor 1α (*HIF1α*) gene expression is up-regulated in hypoxia. *HIF1α* leads to bile concentration by inhibiting the activity of aquaporin 8 (AQP8) in liver, which is one of the reasons for promoting the occurrence of cholesterol stones (Asai et al., 2017). GD is a digestive system disease, which is largely affected by eating habits. Tibetan people consume less vegetables and fruits in their daily diet, but more beef and mutton. They drink butter tea and sweet tea, but less drinking water. Their lifestyle is quite different from that of people in other regions of China.

With the development of sequencing technology, the genetic characteristics of many complex diseases have been gradually revealed. Approximately 25% of the risk of cholelithiasis can be explained by genetics (Katsika et al., 2005). Mutations in some lithogenic genes may be the cause of gallstone formation. Common lithogenic genes include liver cholesterol transporter ATP binding cassette subfamily G member 5 (*ABCG5*) (Di Ciaula et al., 2018), *ABCG8* (Lammert et al., 2016), ATP binding cassette subfamily B member 4 (*ABCB4*), and UDP glucuronosyltransferase family 1 member A1 (*UGT1A1*) (Sun et al., 2021). In addition, other genes related to GD include *CYP7A1*, *GCKR*, *SULT21*, *TM4SF4*, *TTC39B*, etc. (Rebholz et al., 2018).

However, previous studies mainly focused on one or two genes, or several specific exons. Few studies systematically investigated the relationship of genes in the cholesterol metabolism pathway and gallbladder diseases. Here, by targeting all genes in cholesterol metabolism pathway, we explored the relationship of polymorphic loci in cholesterol metabolism pathway and gallstone in the Tibetan populations.

## Materials and Methods

### Study Subjects

A total of 184 subjects were selected from the Tibetan population. Among them, 132 were gallstone patients and 52 were health controls. Gallstone was diagnosed with the Doppler ultrasound of fasting examination by professional doctors. In addition, whole exon sequencing data of 41 healthy Tibetans were downloaded from the CNCB (China National Center for Bioinformation) database (8 samples were downloaded from accession Number PRJCA000600; 33 of 38 samples were downloaded from accession Number PRJNA382306, 5 Sherpa were excluded). This study was approved by the ethics committee of Xizang Minzu University, and all subjects signed informed consent.

### DNA Extraction

3 ml peripheral venous blood were collected from each subject, and was placed in an anticoagulant tube containing ethylenediaminetetraacetic acid (EDTA). DNA was extracted using a blood genomic DNA extraction kit. The concentration, purity, and quality of extracted DNA were examined, and only qualified samples were stored in −20°C refrigerator.

### Genotyping and Quality Control

Genes located in the cholesterol metabolism pathway were sequenced in all samples by the next-generation sequencing method. Single nucleotide polymorphisms (SNPs) calling were implemented by using the standard GATK pipeline (Genome Analysis ToolKit, v4.0.4.0). The criteria for SNPs quality control were: 1) missing rate <0.05; 2) minor allele frequency (MAF) > 0.01; 3) *p* value >0.001 in Hardy-Weinberg equilibrium (HWE) test.

### Statistical Analysis

SPSS v23.0 statistical software was used for data analysis and processing. Genetic association analysis was performed using the Plink software (Purcell et al., 2007). Principal Component Analysis (PCA) was performed by using Smartpca software. After adjusting different numbers of eigen vectors, Q-Q (quantile-quantile) plot and inflation factor were used to evaluate the impact of population heterogeneity on the association results. Association analyses were implemented by using logistic regression under the additive assumption. Manhattan plot was depicted by using R software. Expression Quantitative Trait Locus (eQTL) analyses were implemented in GTEx database. Multiple tests were adjusted by the False Discovery Rate (FDR) method and *FDR-P* < 0.2 was used as the criteria of significance.

## Results

### Sample Population

**FIGURE 1:**
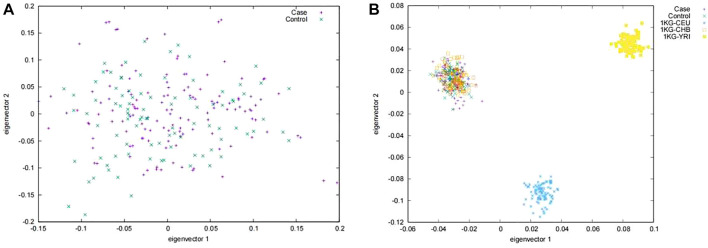
Principal component analysis in **(A)** the Chinese Tibetan population and **(B)** the reference sample in the international 1,000 genomes project. Case, Patients with gallstones; Control, Healthy control population; CEU, Utah Residents (CEPH) with Northern and Western European Ancestry; CHB, Han Chinese in Beijing, China; YRI, Yoruba in Ibadan, Nigeria.

A total of 225 Tibetans were included in this study. Of which, 132 were gallstone patients and 93 were healthy controls. No abnormal deviation was found between the case group and the control group in the PCA analyses (Figure 1A). Further, by combing our subject with the subjects of the International 1000 Genomes Project (Auton et al., 2015), we found that our subjects only aggregated with Han Chinese in Beijing (CHB) population, which indicated that no obvious population heterogeneity were found in our study (Figure 1B).

### Sequencing Region Information

Target sequencing includes 1,042 loci regions of 508 genes in the cholesterol metabolism pathway. The basic information and location of these regions were summarized in Supplementary Table S1. By using the GATK analysis pipeline, a total of 2,401 SNPs were obtained. And, after quality control, 2,011 high-quality SNPs were finally obtained for subsequent analysis.

### The Overall Association Analysis

**FIGURE 2:**
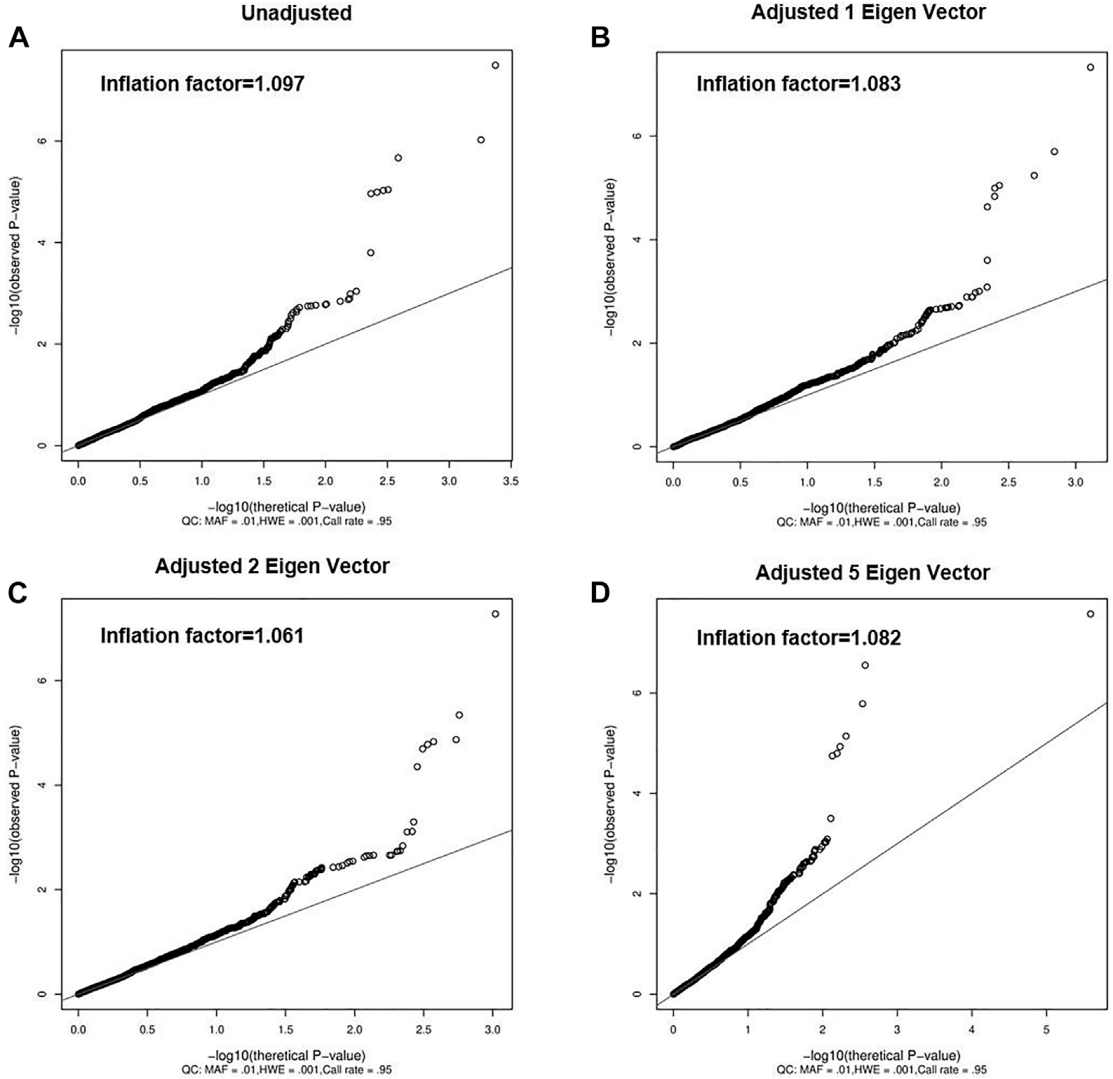
Quantile-Quantile Plot of gallstones. **(A)** does not correct the eigen vector, **(B)** correct for the first eigen vector, **(C)** correct for the top 2 eigen vectors and **(D)** correct for the top 5 eigen vectors. Solid lines indicate the null-hypothesis.

**FIGURE 3:**
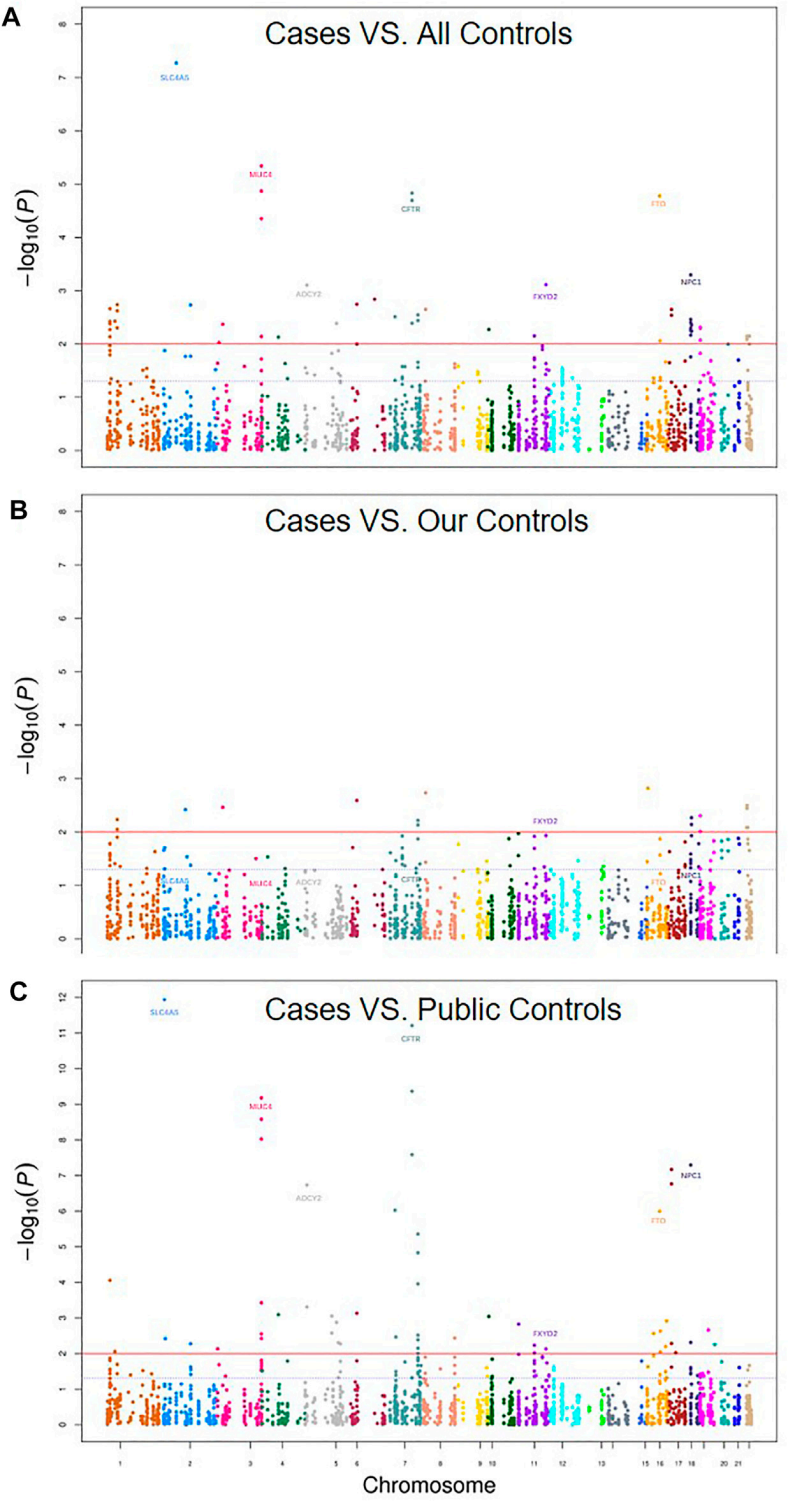
Manhattan Plot of gallstones for **(A)** Cases vs. all controls (our 52 controls +41 public controls), **(B)** Cases. our 52 controls and **(C)** Cases vs. 41 public controls. The plot shows −log_10_ *p* value for each SNP against the chromosomal location. The *x*-axis represents chromosomal position and the *y*-axis shows −log_10_ *p* values. The blue dashed line indicates the *p* value of 0.05 while the solid red line indicates the *p* value of 0.01.

Although the previous PCA analyses did not find obvious population heterogeneity (Figure 1), we still adjusted the top eigen vectors in the following association analyses. By depicting Q-Q plots and calculating the inflation factors, we found that the association result after adjusting top two eigen vectors have the smallest inflation factor (Figure 2). Thus, in the following association analyses, top two eigen vectors were adjusted. Then, by evaluating the association of all 2,011 SNPs with gallstone, we found that 10 SNPs were significantly associated with Gallstone (*FDR-P* < 0.2, Figure 3A and Table 1). All 2,011 loci are listed in Supplementary Table S2.

**TABLE 1:** Overall and subgroup analyses for SNPs which were significantly associated with gallstone (FDR-P < 0.2).

SNP	Chr	BP	Gene	MA^a^	Case genotype			Control genotype			OR (95% CI)^c^	*p* ^c^
					mm^b^	Mm^b^	MM^b^	mm^b^	Mm^b^	MM^b^		
Cases vs. All controls												
SNV05997	2	74,450,135	SLC4A5	G	0	108	24	0	42	51	5.43 (2.95–9.98)	5.30E-08
rs80145081	3	195,510,732	MUC4	A	0	103	25	0	43	47	4.19 (2.27–7.73)	4.56E-06
rs80005560	3	195,510,718	MUC4	T	0	103	28	0	44	49	3.78 (2.08–6.87)	1.35E-05
rs79074685	7	117,188,850	CFTR	T	0	11	121	0	31	62	0.19 (0.09–0.4)	1.47E-05
rs748546375	16	53,913,912	FTO	C	0	84	48	0	31	62	3.46 (1.97–6.1)	1.67E-05
rs201880593	7	117,188,750	CFTR	T	0	7	125	0	27	66	0.14 (0.06–0.35)	2.02E-05
rs142559357	3	195,510,707	MUC4	G	0	103	28	0	45	46	3.49 (1.92–6.36)	4.44E-05
rs750769471	18	21,120,382	NPC1	G	0	95	36	0	44	49	2.74 (1.55–4.84)	5.05E-04
**rs869789**	**11**	**117,691,386**	**FXYD2**	**A**	**15**	**57**	**60**	**3**	**28**	**62**	**2.26 (1.41–3.64)**	**7.72E-04**
rs4072341	5	7,757,705	ADCY2	C	1	12	115	5	20	65	0.33 (0.17–0.63)	7.87E-04
Cases vs. Our control												
SNV05997	2	74,450,135	SLC4A5	G	0	108	24	0	42	10	1.06 (0.46–2.42)	8.96E-01
rs80145081	3	195,510,732	MUC4	A	0	103	25	0	38	11	1.11 (0.49–2.50)	8.02E-01
rs80005560	3	195,510,718	MUC4	T	0	103	28	0	38	14	1.23 (0.58–2.63)	5.90E-01
rs79074685	7	117,188,850	CFTR	T	0	11	121	0	2	50	2.46 (0.52–11.71)	2.60E-01
rs748546375	16	53,913,912	FTO	C	0	84	48	0	28	24	1.49 (0.77–2.87)	2.34E-01
rs201880593	7	117,188,750	CFTR	T	0	7	125	0	3	49	0.97 (0.24–3.95)	9.66E-01
rs142559357	3	195,510,707	MUC4	G	0	103	28	0	38	14	1.23 (0.58–2.63)	5.92E-01
rs750769471	18	21,120,382	NPC1	G	0	95	36	0	38	14	0.91 (0.44–1.90)	7.99E-01
**rs869789**	**11**	**117,691,386**	**FXYD2**	**A**	15	57	60	3	14	35	**2.08 (1.18–3.67)**	**1.17E-02**
rs4072341	5	7,757,705	ADCY2	C	1	12	115	0	3	47	1.72 (0.50–6.00)	3.92E-01
Cases vs. Public control												
SNV05997	2	74,450,135	SLC4A5	G	0	108	24	0	0	41	—	2.88E-12
rs80145081	3	195,510,732	MUC4	A	0	103	25	0	5	36	29.59 (10.10–86.72)	6.61E-10
rs80005560	3	195,510,718	MUC4	T	0	103	28	0	6	35	22.33 (8.03–62.07)	2.63E-09
rs79074685	7	117,188,850	CFTR	T	0	11	121	0	29	12	0.03 (0.01–0.09)	6.11E-12
rs748546375	16	53,913,912	FTO	C	0	84	48	0	3	38	21.98 (6.37–75.84)	1.01E-06
rs201880593	7	117,188,750	CFTR	T	0	7	125	0	24	17	0.04 (0.01–0.11)	4.29E-10
rs142559357	3	195,510,707	MUC4	G	0	103	28	0	7	32	17.39 (6.56–46.12)	9.43E-09
rs750769471	18	21,120,382	NPC1	G	0	95	36	0	6	35	14.24 (5.48–37.03)	5.08E-08
**rs869789**	**11**	**117,691,386**	**FXYD2**	**A**	15	57	60	0	14	27	**2.51 (1.28–4.91)**	**7.47E-03**
rs4072341	5	7,757,705	ADCY2	C	1	12	115	5	17	18	0.12 (0.05–0.26)	1.85E-07

a MA, Minor Allele.

b mm-Minor allele homozygous, Mm-Major and minor allele heterzygous, MM-Major allele homozygous.

c Original *p* values after adjusting top 2 eigen vectors.

Bold values indicates statistical significance in all three cohorts.

### Association Analyses in Subset Datasets

Considering that our controls including two parts: our own 52 subjects and 41 subjects from the public database. We further did subgroup analyses by using our own 52 subjects only and by using 41 public subjects only. We found that the SNP rs869789, which located in the 3′ UTR region of *FXYD2*, were consistently significantly associated gallstone in the subgroup analyses (Figures 3B,C; Table 1). The frequency of A allele in cases was 0.330, which were significantly higher than that in our control (0.192, OR = 2.06, *p* = 9.04 × 10^–3^) and in public controls (0.171, OR = 2.39, *p* = 5.73 × 10^–3^). By implementing eQTL analyses in GTEx, we found that A allele of rs869789 was significantly correlated with a higher expression of *FXYD2* in colon-sigmoid tissue, thyroid tissue, colon-transverse tissue, esophagus-gastroesophageal junction tissue and nerve-tibial tissue (Supplementary Figure S1).

## Discussion

The disease composition of people in the high altitude environment is different from that in other areas, and the incidence of gallbladder diseases is increasing year by year. Cholesterol gallstones are the most common type of gallstones, and the change of cholesterol homeostasis may be one of the causes of gallstones. Vegetarians can reduce the risk of gallstones by reducing cholesterol levels in the body (Chang et al., 2019). Interestingly, another prospective cohort study pointed out that vegetarians had a significantly higher risk of symptomatic gallstone disease than non-vegetarians after adjusting for risk factors, including body mass index (McConnell et al., 2017). We speculated that this might be related to the difference in gene expression in the cholesterol metabolism pathway. There are many genes in the cholesterol metabolism pathway, which directly or indirectly affect the metabolism level of cholesterol in the body, which may have some unknown correlation with the occurrence of GD. In this study, through the detection of cholesterol metabolism pathway genes in Tibetan people living in Tibet, it was found that ten SNPs loci were associated with gallstone, of which SNP rs869789 was most significantly associated with gallstone in Tibetan people. We analyzed the information of 10 SNPs screened. These loci are located in seven different genes, including *SLC4A5*, *MUC4*, *FTO*, *NPC1* and *FXYD2* genes that may increase the risk of gallstone in the Tibetan population, while *CFTR* and *ADCY2* genes that reduce the risk of gallstone in the Tibetan population.

The Solute carrier family 4 Member 5 (*SLC4A5*) gene encodes Na^+^-HCO_3_^−^ cotransporter, which plays a role in regulating sodium and bicarbonate transport and affects intracellular, extracellular, interstitial and ultimately plasma Ph (Felder et al., 2016). The sodium bicarbonate cotransporter *SLC4A5* plays an important role in the recovery of cerebrospinal fluid pH during hypercapnia-induced acidosis, which can protect the brain from acid damage (Christensen et al., 2018). In gallbladder diseases, some elements in bile (such as Ca^2+^, Fe^3+^, Cu^2+^) are very significant in the development of GD (Khan et al., 2017). The higher density and pH value of bile and the higher concentration of transition elements may be important factors for the formation of different types of GD. In a population survey, it was found that people with hypertension have a higher risk of gallstones than the general population, which may be related to the mutation of *SLC4A5* gene loci (Song et al., 2020). A number of studies have pointed out that there is a genetic association between *SLC4A5* gene polymorphism and hypertension (Parker, 2018; Barbuskaite et al., 2020). A healthy diet can reduce the risk of gallstone disease while preventing high blood pressure (Wirth et al., 2018). Our results showed that SNV05997 in *SLC4A5* gene is a risk factor for GD in Tibetan people, and it is speculated that the dietary structure of Tibetan people may induce the mutation of *SLC4A5* gene, and changing diet may play a certain role in the prevention of gallstone.

The microflora of the gastrointestinal tract and biliary tract are involved in the formation of bile, and are related to various complications of gallstone (such as acute and chronic cholecystitis, cholangitis, pancreatitis, etc.), and also related to dysbacteriosis. Studies have shown that oral pathogenic bacteria affect gallbladder movement and the expression of mucin genes (*MUC1*, *MUC3*, and *MUC4*) through the immune regulation mechanism, regulate cholesterol metabolism and promote the formation of gallstone (Grigor’eva and Romanova, 2020). Hu et al. (Hu et al., 2021a) analyzed the gallbladder tissue and bile samples of patients with gallstones and found that the Gram-positive microflora and MUC4 protein in the bile of patients with gallstones were positively correlated with the calcification of cholesterol stones, and there was a synergistic effect. *MUC* family genes are a contributing factor to the formation of cholesterol crystal nuclei. Cholesterol stones and gallbladder infections are associated with increased expression of mucin genes *MUC3* and *MUC5B* (Yoo et al., 2016). In the study of mouse models, it was found that the mRNA expression levels of *MUC2*, *MUC5AC*, *MUC5B* and *MUC6* genes in cholesterol gallstone mice were significantly increased (Kim et al., 2012). The increase of MUC1 mucin in the gallbladder epithelium can promote the absorption of cholesterol in mice and inhibit the movement of the gallbladder, thereby promoting the formation of gallstones (Wang et al., 2006; Chuang et al., 2011) found *MUC1* and *MUC2* gene loci significantly associated with gallstones in Chinese males. We found that *MUC4* gene rs80145081, rs80005560 and rs142559357 are risk factors for GD in the Tibetan population of China, which is an effective supplement to the susceptibility site of *MUC* family gene in GD, and provides clues for exploring the mechanism of *MUC* family gene co-action to increase susceptibility to gallstones in Chinese population.

The human alpha-ketoglutarate dependent dioxygenase (*FTO*) genes is involved in DNA repair and fatty acid metabolism (Franczak et al., 2018). In mice with fused-toe (Ft) mutation, there is a gene named fat mass and obesity associated *(FTO*) gene, which is the orthologs gene of human, causing obesity (Chen and Du, 2019). In humans, the overexpression of *FTO* gene is manifested in the increase of body mass index (BMI) and basal metabolic rate (BMR) (Shaikh et al., 2021; Stender et al., 2013) found that people with elevated BMI are more likely to suffer from symptomatic gallstone disease by comparing gallstone people with healthy people. Obese people usually show an increase in fasting gallbladder volume and a decrease in postprandial gallbladder emptying, which lead to gallbladder stasis and promote the formation of gallstones (Di Ciaula et al., 2012). *FTO* gene is highly expressed in the hypothalamus, which is a key region regulating feeding and energy consumption (Chen and Du, 2019). It is speculated that the *FTO* gene may have the function of regulating the feeding center. Studies have shown that the increase of abdominal fat mass leads to decreased gallbladder motility and cholestasis. Therefore, factors affecting adipocyte secretion or metabolism may also affect the formation of gallstones (Stender et al., 2013). Gallstones are closely related to obesity. The emptying time of the gallbladder in obese people is similar to that of normal people, while the refilling time of the gallbladder is significantly shortened, showing enhanced enterohepatic circulation dynamics, resulting in a significant increase in blood cholesterol levels and a higher risk of cholesterol stone formation (Kubica and Balbus, 2021). Population studies have shown that *FTO* gene has SNP loci regulating fat metabolism in human body, leading to the occurrence of obesity (Mehrdad et al., 2020; Shaikh et al., 2021). We speculated that obese patients are more prone to GD, which is related to the mutation site of *FTO* gene to a certain extent. In this study, we found that rs748546375 was related to the increased risk of GD in the Tibetan population.

It has been reported that NPC intracellular cholesterol transporter 1 (*NPC1*) gene defect can lead to Niemann-Pick C genetic disease, excessive accumulation of cholesterol *in vivo* and autophagy dysfunction (Ilnytska et al., 2021). Both NPC gene products participate in the same cholesterol transport pathway and perform different but complementary functions, playing a role in transferring cholesterol from the liver to bile and blood (Klein et al., 2006; Hu P. et al., 2021). Also, the mouse animal model showed that *NPC1* gene regulates the formation of gallstones by controlling cholesterol in the liver, suggesting that the expression of *NPC1* gene may be related to the formation of cholesterol stones (Morales et al., 2010). Yuan et al. (Yuan et al., 2005) Screened 149 lipid related genes expressed in gallbladder, including gallstone candidate susceptibility gene *NPC1*. Similarly, our results also found that *NPC1* gene SNP rs750769471 may be related to gallstone.

The proteins encoded by FXYD domain containing ion transport regulator (*FXYD*) gene family belong to a family of small-membrane proteins, which play a key role in the regulation of NA-K-ATPase (Zhao et al., 2020). The activity of NA-K-AtPase depends on phosphatidylserine (PS) and cholesterol. PS and cholesterol bind near the *FXYD* subunit, so the mutation of *FXYD* site may be associated with cholesterol (Habeck et al., 2017). It has been reported that the high expression of *FXYD2* gene is related to poor overall survival, which may cause changes in tumor microenvironment (TME) and lead to malignant tumors (Zhao et al., 2020). Through the detection of gene expression in normal tissues, it was found that *FXYD2* gene was highly expressed in gallbladder, with tissue specificity (Fagerberg et al., 2014). Studies have shown that the region of chromosome 11q22-24 is involved in the occurrence of schizophrenia (Choudhury et al., 2007). *FXYD6* gene mutation may affect the brain region and lead to schizophrenia. Chaumette et al. (Chaumette et al., 2020) found that there are pathogenic missense variants of *FXYD* gene family *FXYD1*, *FXYD6* and *FXYD6-FXYD2 readthrough* in childhood onset schizophrenia. Among them, *FXYD6-FXYD2 readthrough* is a connecting gene, which produces transcripts by binding exons of *FXYD6* and *FXYD2* in the same direction on the same chromosome.

In our study, we found that the *FXYD* gene family SNP rs869789 was significantly associated with gallstones. (Zhang et al., 2010) found no significant relationship between SNP rs869789 in *FXYD* gene family and the occurrence of schizophrenia in Chinese population through sequencing analysis. However, Tauroursodeoxycholic acid (TUDCA), a clinical drug for the treatment of gallstones, can play a neuroprotective role in different brain diseases, protect neurons from neurodegeneration and prevent neuroinflammation and oxidative stress (Cheng et al., 2019; Lu et al., 2018). In the process of atherosclerosis, the expression of *FXYD3* gene was significantly down-regulated, while the expression of *FXYD6* was upregulated (Liu et al., 2007; Dong et al., 2021). Through cell experiments, (Dong et al., 2021) found that lncRNA up-regulated the expression of *FXYD6*, increased the uptake of cholesterol by macrophages, induced the production of inflammatory molecules, and promoted atherosclerosis. *FXYD* family genes may lead to cholesterol aggregation, and gene mutations may lead to excessive cholesterol aggregation and gallstone. The specific mechanism of rs869789 and gallstone should be further studied in cell experiments.

In addition, we found that the SNP locus rs79074685 and rs201880593 of the cystic fibrosis transmembrane conductance regulator (*CFTR*) gene located on chromosome 7 could reduce the risk of GD disease in the Tibetan population at high altitudes. *CTFR* is involved in gastric acid secretion, chemokine signal transduction, bile secretion, and apoptosis (Wu et al., 2020). The absence of *CFTR* affects the apical junction complex of the bile duct epithelium. The mislocalization of *CFTR* in mouse bile duct cells makes mice present a cholestatic phenotype (Fiorotto et al., 2016). *CFTR* located in the bile secretion pathway is involved in the regulation of pH of rat ameloblasts, and then in the development of tooth germ (Yang et al., 2020). *CFTR* may be involved in the formation of calcification, leading to the formation of GD. Some SNP mutations in the *CFTR* gene may have a certain impact on this process. Cystic fibrosis (CF) is an autosomal recessive genetic disease, which is caused by *CFTR* gene mutation leading to CFTR protein misfolding, defective transport, and impaired function (Kumar et al., 2021). *CFTR* gene mutation leads to high oxaluric acid urine, acidic urine, and low urine volume, making CF patients more susceptible to urinary calculi (Wright et al., 2021). We speculate that mutations in the *CFTR* gene can cause changes in various urine indicators, and there may also be mutations that have a certain effect on the biochemistry of bile.

Cholesterol stones may cause obesity and dyslipidemia, and adipocyte dysfunction may lead to gallstone (Han et al., 2019). Our study showed that the alteration of rs4072341 in adenylyl cyclase 2 (*ADCY2*) gene also reduced the occurrence of gallstones. In addition, (Daily et al., 2019) found that *ADCY2* rs326149 affects subcutaneous fat, which is positively correlated with the amount of subcutaneous fat, resulting in a high level of serum high-density lipoprotein cholesterol level. Previous studies have shown that *ADCY2* gene is expressed in the brain and encodes a membrane-binding enzyme for the synthesis of cyclic adenosine monophosphate (cAMP). Hypoxic environment causes changes in reactive oxygen species (ROS) levels and also activates cAMP (Wang et al., 2021). cAMP is involved in the regulation of preadipocyte differentiation (Yadav and Jang, 2021). cAMP signaling is associated with adipogenic differentiation of bone marrow mesenchymal stem cells (Chen et al., 2021). Therefore, we speculate that *ADCY2* gene may be involved in adipocyte differentiation and thus affect the formation of gallstones.

As a case-control study, we tried our best to match the information of the two groups of subjects. However, in the process of sample recruitment, due to the difference in population distribution in high-altitude areas, the two groups were not completely equal. Therefore, through the application of public databases, we have improved relevant data while saving biological information resources, making this experimental analysis more reliable. Meanwhile, this study was conducted in a high-altitude area in Tibet, China. People living in the same environment had roughly the same diet structure and living habits, so it was a targeted study on the genetic mechanism of GD without the interference of environmental factors.

## Conclusion

In summary, this study explored the genetic mechanism of gallstones in high altitude populations based on restricted environment and diet, which is the first systematic sequencing analysis of genes in the cholesterol metabolism signal pathway in the Tibetan gallstone population. We found that ten independent SNPs were located in different positions, especially *FXYD2* gene rs869789 was significant in all groups. These gene loci are associated with gallstones and are worthy of in-depth follow-up research, guiding the health of people living in the plateau.

## Data Availability

The data presented in the study are deposited in the Genome Sequence Archive (Genomics, Proteomics and Bioinformatics 2021) in National Genomics Data Center (Nucleic Acids Res 2022), China National Center for Bioinformation/Beijing Institute of Genomics, Chinese Academy of Sciences (GSA-Human: HRA002309) that are publicly accessible at https://ngdc.cncb.ac.cn/gsa-human.
