# Supplementary material for: Association Study Between Polymorphic Loci in Cholesterol Metabolism Pathway and Gallstone in the Tibetan Population
Source: Front Genet. 2022 May 16;13:902553. doi: 10.3389/fgene.2022.902553 (PMC9149373; doi:10.3389/fgene.2022.902553)
Supplement: Supplementary file 1 [file DataSheet1.DOCX]

Supplementary Material

## Supplementary Figure


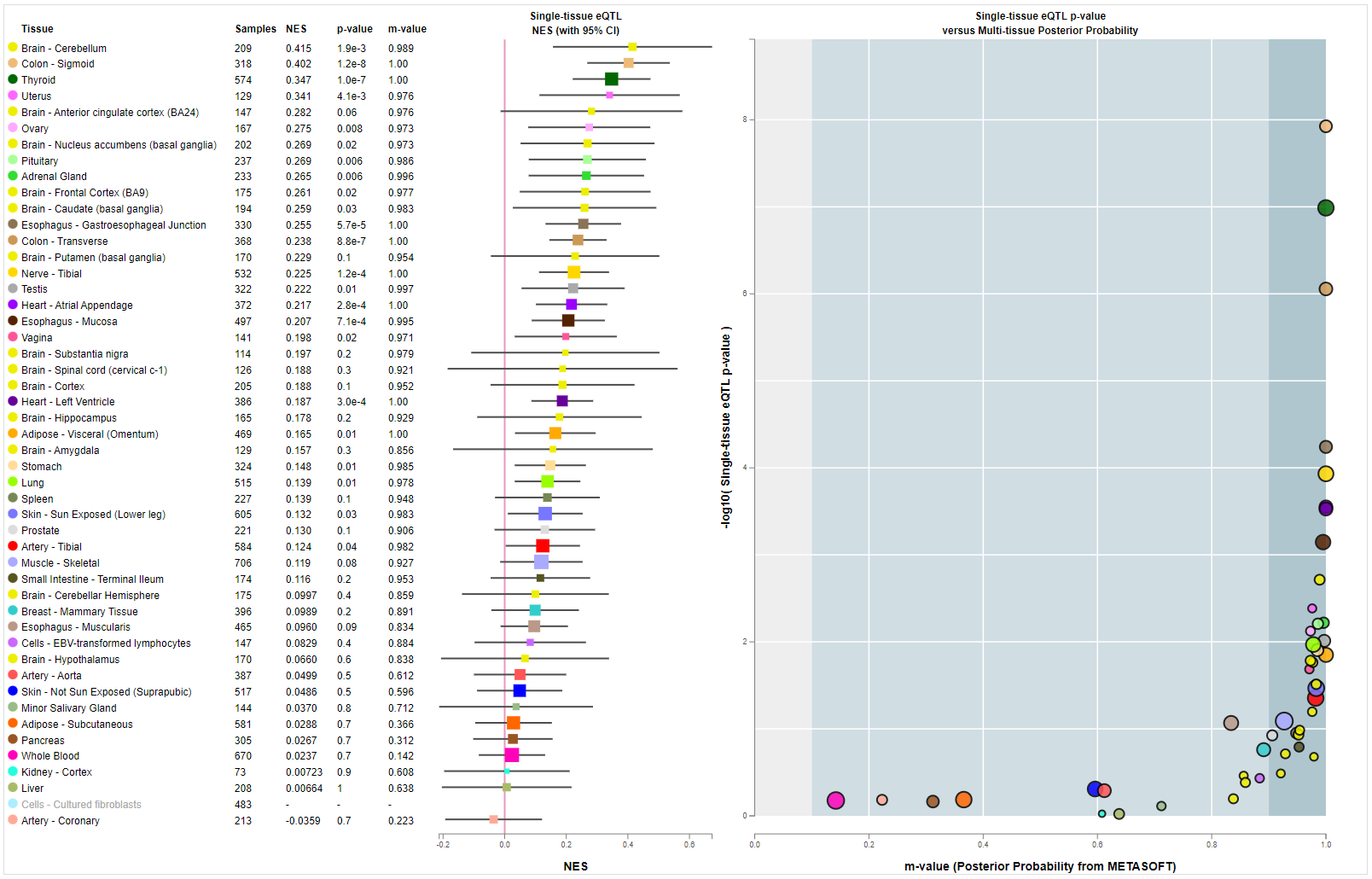


**Supplementary Figure 1.** rs869789 in *FXYD2* gene by Expression Quantitative Trait Locus (eQTL) analyses.

## Supplementary Tables

**Supplementary Table 1.** Genes in our panel.

See the excel file for the table content.

**Supplementary Table 2.** Association of all SNPs with gallstone.

See the excel file for the table content.
