# Supplementary material for: Association Study Between Polymorphic Loci in Cholesterol Metabolism Pathway and Gallstone in the Tibetan Population
Source: Front Genet. 2022 May 16;13:902553. doi: 10.3389/fgene.2022.902553 (PMC9149373; doi:10.3389/fgene.2022.902553)
Supplement: Supplementary file 2 [file Image1.pdf]

Supplementary Material

1.1 Supplementary Figure

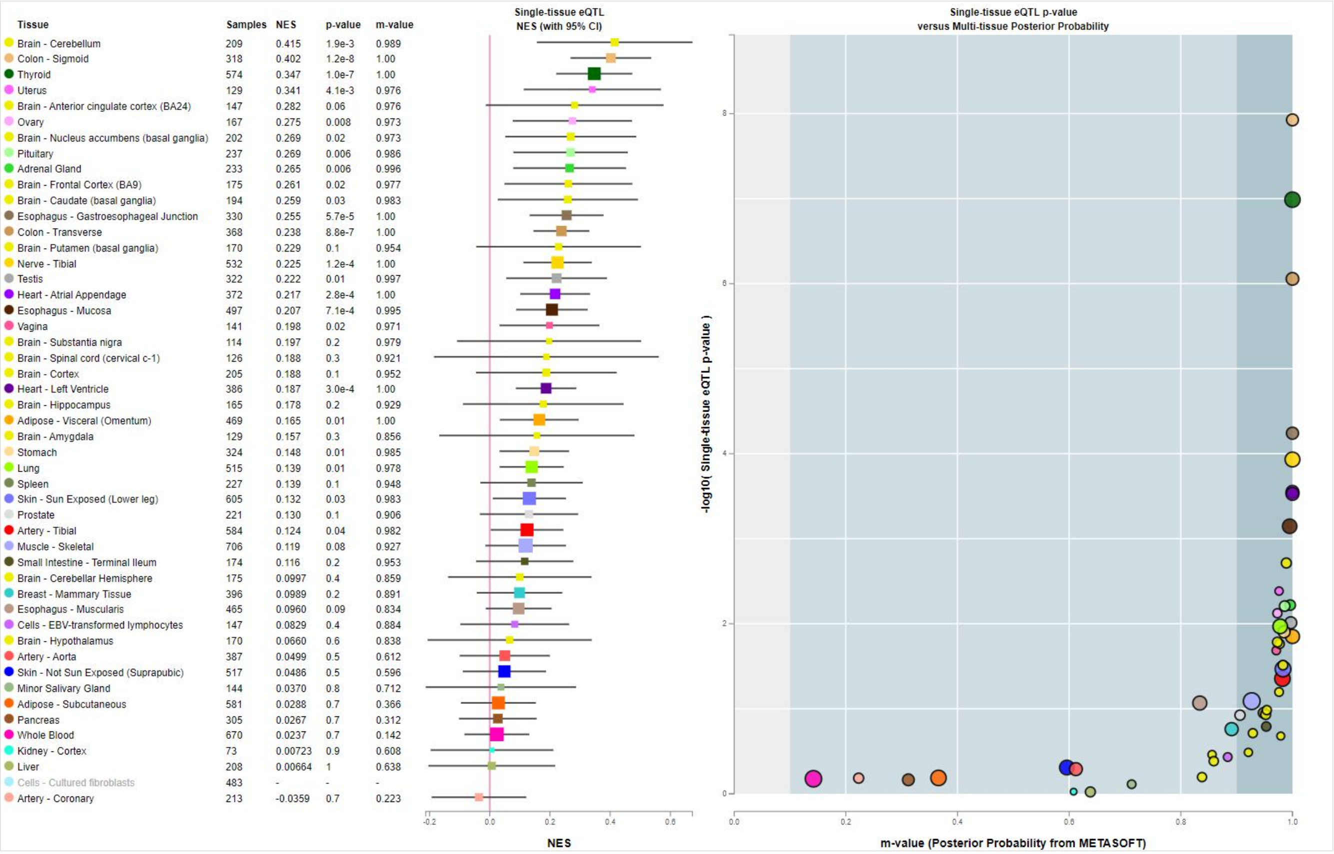

Supplementary Figure 1. rs869789 in *FXYD2* gene by Expression Quantitative Trait Locus (eQTL) analyses.

1.2 Supplementary Tables

Supplementary Table 1. Genes in our panel.

See the excel file for the table content.

Supplementary Table 2. Association of all SNPs with gallstone.

See the excel file for the table content.
